# Developing a Sustainable Long-Term Ageing Health Care System Using the DANP-mV Model: Empirical Case of Taiwan

**DOI:** 10.3390/ijerph16081349

**Published:** 2019-04-15

**Authors:** Pei-Jian Lin, Yih-Chearng Shiue, Gwo-Hshiung Tzeng, Shan-Lin Huang

**Affiliations:** 1 Department of Business Administration, National Central University, No. 300, Zhongda Rd., Zhongli District, Taoyuan 32001, Taiwan; Lcp313419@gmail.com (P.-J.L.); ycs@mgt.ncu.edu.tw (Y.-C.S.); 2 Graduate Institute of Urban Planning, College of Public Affairs, National Taipei University, 151, University Rd., San Shia District, New Taipei 23741, Taiwan; ghtzeng@gm.ntpu.edu.tw; 3 Department of Tourism Management, Tourism School, Sanming University, 25, Jingdong Rd., Sanyuan District, Sanming 365004, China; 4 National Park Center, Sanming University, 25, Jingdong Rd., Sanyuan District, Sanming 365004, China

**Keywords:** baby boomers, sustainable system, long-term ageing health care, DANP-mV model, continuous improvement strategy

## Abstract

Most of the baby boomers born after the Second World War (WWII) have passed the age of 65, meaning they have gradually lost their social functions and positions, and are facing the need for care. In Taiwan, the lack of a long-term care mechanism is having a certain degree of impact on society as a whole, and thus, it is important to have a mechanism to take care of the elderly. In order to make this system sustainable, sufficient funds and continuous improvement are important factors. In the past, in order to avoid the illegal transfer of benefits, the social welfare mechanism avoided the use of for-profit organizations. However, as the economic environment declines, the role of for-profit organizations should be considered. This study defines the long-term ageing health care system using five major dimensions and 20 criteria. The DANP-mV model was used to analyze Taiwan’s current system and identify problems, and then to develop a continuous improvement strategy from the perspective of the source of the problem in order to improve long-term ageing health care.

## 1. Introduction

After the Second World War, all walks of life were faced with the need for revival and development. People born in this period are called the post-war baby boom generation (the baby boomer generation), and they contributed greatly to the reconstruction of the overall environment and economic system after the war [1]. Most of them are now over 65 years old, and have gradually lost their social position and function [2]. For the elderly, the lack of an effective care mechanism shows a lack of respect, and has a negative impact on the development of communities. As people age, their physiological functions gradually recede, which is often associated with disease [3]. Whether due to deterioration or chronic disease, the family burden to take care of seniors will increase [4]. Therefore, the development of a sustainable long-term ageing health care system can enable ageing people to enjoy a dignified and healthy life, and can significantly reduce the burden of raising families; thus, the labor force can once again be invested in the production system, which will promote social development.

The mechanism of long-term ageing health care has been widely discussed. Scholars in the field of nursing and elderly care have engaged in in-depth studies, and put forward many ways to maintain aged people’s physical and mental health development, which are integrated with social welfare from the government to provide the corresponding support [5]. More and more scholars believe that the problem of ageing care must apply integrated thinking; therefore, the concept of medical integration was put forward [6]. The core of this concept is integration into different systems, from disease prevention, medical treatments, and follow-up maintenance to physical and spiritual cares and concerns. In studies of integrated medical treatment, health care for the elderly is regarded as a long-term and comprehensive system that must be regulated in a holistic manner, from health maintenance to disease treatment to physical and mental care [7]. Therefore, long-term care systems for older adults must be designed with a comprehensive perspective. However, the sustainability of system “funding” is a very important factor, as sources of funding can be diversified. In the past, the sources of funds for social welfare systems were mostly from the government and non-profit organizations, in order to avoid the problem of benefit transfer. While this system currently has sustainability, if is faced with the decline of fiscal revenues, and the sustainability of this system will face great challenges and threats; therefore, the transparent incorporation of private sector investment plays an important role in the sustainability of the system; however, there has been a lack of such participation. In consideration of private enterprise, this study examines the health index of of the elderly long-term care system architecture, and through the DANP-mV model, attempts to understand the problems in Taiwan’s long-term ageing health care system and identify the core control factors from a systemic point of view. Thus, the root problems are presented by the factors discussed in the improvement strategy, in order to allow the system to achieve sustainable development.

Compared with other countries, Taiwan’s population is ageing rapidly, and in 2018, the population structure reached the level of an aged society. Not only is the number of elderly rapidly increasing, young and middle-aged ethnic groups are also facing the problem of late marriage and fewer children, which leads to a shortage of the overall workforce and a rapid rise of the dependency ratio of aged social problems, meaning the overall population structure is developing in a negative way. Under the pressure of this extreme problem, it has become an urgent task to develop a long-term health care system with sustainable management strategies for ageing people. Therefore, the purpose of this study is to determine the core control factors and understand the current issues of the system through influential relationships, and then propose a set of sustainable long-term ageing health care system. However, in a comprehensive system, all the original independent operating systems are bound to have a high degree of influence, and thus research methods with independent assumptions will inevitably lead to a certain degree of bias in the research results. The main effect of DEMATEL (Decision making trial and evaluation laboratory), as based on the analytic network process (DANP) with a modified VIKOR (DANP-mV) model, is to put forward root improvement strategies, which are compatible from a systematic point of view, by clarifying the influential relationships and problems among the factors [8]. The DANP-mV model can be used to present the most appropriate method for this study, investigate the problems of the long-term ageing health care system in Taiwan, and discover the core control factors according to a systematic perspective [9]. Through discussions of these factors, a fundamental improvement strategy is proposed to create a sustainable system.

The remainder of this paper is organized, as follows: Section 2 introduces the index framework and research process of the long-term ageing health care system; Section 3 discusses the current status and improvement strategy of the long-term ageing health care system in Taiwan; Section 4 offers conclusions.

## 2. Long-Term Ageing Health Care Developmental System

This section, which is divided into three sections, establishes a developmental system for sustainable long-term elderly health care. Section 1 discusses the impact indicators of long-term elderly care, Section 2 uses the pre-test results to establish the framework indicators, and Section 3 explains how the DANP-mV model can be used to analyze data and propose sustainable improvement strategies.

### 2.1. Impact Indicators of Long-Term Ageing Health Care

At the end of the 20th century, more people around the world began to be affected by ageing, with many countries experiencing certain social problems caused by ageing. The rapid growth of the elderly population has placed a heavy burden on medical-care and health-care systems. The responsibility rests with the government to actively manage the social problems associated with ageing by understanding the needs of the elderly population and establishing a solid foundation for the ageing health care service system [10]. Access to certain living conditions and human dignity must be the basis on which the needs of the elderly population are assessed. Therefore, developing and maintaining a long-term ageing health care system is crucial for the government to implement long-term care policies; however, such a system can be affected by many external factors. As the professional health-care workforce is sufficiently large for the smooth completion of ageing health care tasks, it has profound influence on the long-term care system [11,12,13]. As the adequacy of funds drastically affects the adequacy of the workforce, the relationship of the system to funding sources also significantly affects the maintenance and development of the ongoing long-term ageing health care system [14]. Science and technology can effectively integrate cross-platforms and cross-regional case-management health data to solve the problem of conventional manpower not being easily implemented. Therefore, the application of technology is essential to increasing the integration efficiency of the long-term ageing health system [15]. However, the long-term ageing health system includes various medical services, such as nursing and treatment, as well as non-medical services, such as house-cleaning, laundry, and food delivery, and each individual has different care needs. Therefore, the long-term ageing health care system must factor in the necessity of various services [16]. Integration of the health-care system can add convenience to the lives of the ageing population, and the preventive effect of health promotion can effectively reduce medical dependence, which will enable older adults to live healthier and more dignified lives [17]. Since the functioning of modern society depends on the existence of certain norms and systems, such as funding sources, working hours, and wages, as well as the use of new technologies, it is necessary to design relevant norms and systems to make long-term care systems for older adults operate more efficiently [18]. Therefore, this paper proposes that five dimensions can be identified as factors contributing to the development of a long-term care system for the elderly population: workforce, source of funding, technology application, service nature and health-care system, and norms. The interrelationship of these dimensions is also fundamental for the sustainable development of the system. However, as each dimension has its own independent features and functions, the following subsections provide explanations for each individual dimension.

#### 2.1.1. Workforce

A strong workforce is essential to long-term ageing health care, and thus this dimension consists of four criteria: workforce matching platform, specialization level, workforce supply capacity, and labour service flexibility [19].

● Workforce matching platform

A long-term elderly-care system must include a labor supply and a match-filtering platform, as these focus on providing services to balance labor and demand [20], while an imbalance between supply and demand can severely waste resources and cause disintegration of the care-service system, which indirectly burdens the family. Therefore, media platforms must provide a powerful matching function to connect the service personnel with those in need of care to effectively operate the care system [21].

● Specialization level

The specialization level includes two specializations. One is the expertise of the health-care-system provider; the health-care-service industry is different from other service industries, as the service target is primarily unwell, elderly individuals, thus, the personnel engaged in this industry must have sufficient medical-care knowledge [20]. The second is professional service behavior and attitude. As the process of providing health services to elderly individuals has characteristics that vary from person to person, service personnel must possess sufficient professional service behaviors and attitudes to enable them to appreciate the unique characteristics of such a group of individuals. Accordingly, members of the workforce can develop appropriate treatment methods to encourage elderly individuals to develop a sense of satisfaction and trust in the health-care system [4].

● Workforce supply capacity

Service manpower is a crucial driving force behind the long-term ageing health care system. Shortages in the labor market are the result of unfair societal treatment (such as low wages) of individuals working in human services. Moreover, those individuals who have trained to provide health-care services do not want to leave the industry because of an increase in the ageing population; therefore, the shortage of manpower causes problems in the operation of care services, which affects the operations of the entire system [22].

● Labor service flexibility

Labor service flexibility refers to the number of projects that service labor can provide within the service time. A flexible scheduling environment affects the service performance of cost and demand, whereas greater scheduling flexibility produces benefits that depend on specific circumstances [23]. As neither the individual providing service labor nor the recipient of the service is an average individual, each worker will differ regarding their professional ability. Therefore, labor service flexibility can increase the efficiency of the overall system application [20].

#### 2.1.2. Source of Funding

As the cost of medical-care services is determined according to variations in individual needs and medical conditions, and because service projects requiring higher professional degrees correlate to higher costs, variations also exist in the economic conditions of each provider [14]. Therefore, the sources of funding for the effective operations of the health-care system must be explored. This study divides the sources of long-term ageing health care funding into five sections: 1. government subsidies, 2. stable pensions, 3. social insurance, 4. commercial insurance, and 5. public welfare invested donations.

● Government subsidies

Long-term ageing health care is a part of the social welfare system [24]. The government aims to provide more vulnerable elderly individuals with improved care to compensate for the gap between the rich and the poor. Therefore, to support system operations, the government regularly invests financial revenues into the system [25]; conversely, health care in Germany is jointly undertaken by the central and local governments at all levels [24].

● Stable pension

The ability of the elderly to pay for the long-term care system is crucial to the maintenance of the entire system. Stable economic income not only enables elderly individuals to pay their own service expenses, it also enables them to attain inner security and happiness. The primary source of economic income for elderly adults is usually their own retirement pension [26]. In other words, pensions determine the ability of elderly individuals to pay for long-term care, which affects the operations of the entire system [27].

● Social insurance

An insurance system can be interpreted as a risk-sharing mechanism for seniors [28]. Social insurance refers to the guarantee that the government maintains a basic long-term health-care system (social welfare and long-term care systems) [17,29]. As this is a basic safeguard system, the government is responsible for reducing the negative effects of the economic changes that affect the financial influences, in order to maintain a long-term health-care system [24,30].

● Commercial insurance

As everyone has different needs, commercial insurance provides an additional safeguard to the long-term health-care system. In the long run, increasing the use of commercial insurance may effectively reduce the burden of national tax subsidies [25].

● Public welfare invested donation

Rising awareness of corporate social responsibility means that philanthropy based on the practice of providing charitable donations could not only provide enterprises with competitive advantages, and could also expand its benefits to include the encouragement of related charitable actions [31,32]. Therefore, the investment of enterprise resources in public welfare donations is a new concept that can also be regarded as a type of enterprise investment [33]. Moreover, in the public welfare system immediate compensation is in high demand.

#### 2.1.3. Application of technology

Long-term health-care industries often require a large workforce and cross-platform collaborative professional services. Therefore, the application of technology in the health care industry can effectively provide service workers with a more accurate method to accomplish complex and large-scale target management activities, and enable elderly individuals and health-industry workers access to more comprehensive protection. The protective dimensions afforded by the introduction of technological applications to health care can be divided into three categories: smart physiological monitoring devices, intelligent case care systems, and living-environment monitoring systems.

● Smart physiological monitoring facility

A smart physiological monitoring facility can integrate wireless health-monitoring equipment and a base medical system, which would enable systems in different domains to collaborate and simultaneously store various types of data in different domains [15], thereby, effectively reducing operating costs and maximizing the capabilities of long-term care systems [34].

● Intelligent case care system

Health-care workers are often confronted with different long-term care requirements, depending on the needs of the particular individual in need of care. Therefore, among the ageing population, the problem of diverse demands can be solved using technology to enable elderly individuals to attain an improved quality of life and comprehensive protection. This application of technology is referred to as an intelligent case care system [10].

● Living-environment monitoring system

A living-environment monitoring system is a system that integrates different system platforms and intelligent communication devices [15], which primarily monitors the daily living environment of elderly individuals to provide them with direct relief and assistance in the event of an emergency [35].

#### 2.1.4. Service Nature and Health-Care System

Service is the essence of a health-care-service system. The primary purpose of the system is to enable all elderly people to live a dignified and healthy life, in addition to being able to live in excellent health in a safe environment. Therefore, this paper discusses the integration of “residential services,” “case care services,” “integration of health,” and “health promotion.”

● Residential services

Due to the gradual decline in physical fitness associated with ageing, services for household cleaning, laundry, food, and beverage have become increasingly crucial. Therefore, residential services are designed to solve problems in the daily lives of elderly individuals who live at home to improve their health status and indirectly promote their healthy lifestyles [17].

● Case care services

As each elderly person differs regarding their individual characteristics and conditions, case care services can ensure higher quality health-care services [10,36].

● Integration of health

In a long-term health-care system, a type of compound care is often required by those in need of care. To support such care, medical and care units must integrate the entire system to utilize resources and increase efficiency to meet the needs of those in need of care [22,37].

● Health promotion

The main goal of health promotion is to replace treatment with prevention, the hope being that elderly individuals can engage in non-medical treatment of diseases before symptoms appear [10,38]. Therefore, this idea is advocated by modern medical units as the preferred method for promoting health in elderly individuals [39].

#### 2.1.5. Norms

In addition to the various rules and policies, law provides the basis for social work, thus development in all industries is the development of these norms [40]. This study is based on four aspects: institutional setting, operations management, financial investment, and resource investment. Therefore, the dimensions of a norm include institutional setting standards, operational specification, financial specification, and enterprise resource input specification.

● Institutional setting standards

Regulations and standards for long-term health-care-service providers are designed to ensure the legitimacy of service providers and enable them to operate normally, stably, and permanently [31,33].

● Operational specification

Through the establishment of industry operational standards, the possibility of human operational errors can be effectively eliminated to improve overall transparency [41]. Therefore, operational specifications play a fundamental role in determining the effectiveness of long-term health-care systems [42].

● Financial specification

Financial specifications refer to the financial transparency, normalization, and institutionalization of laws and regulations. Financial specification provides the entire system with the ability to manage and prevent risks and financial crisis [43].

● Enterprise resource input specification

Enterprise resource input refers to using the concept of corporate social responsibility to provide another fund-injection channel for the health-care-service industry [44]. However, the nature of social welfare units differs from that of commercial units, meaning that the regulations for enterprise resource input can effectively prevent the qualitative change of social welfare units, and thus, fail to initiate progress toward real sustainable development [42].

**Figure 1:**
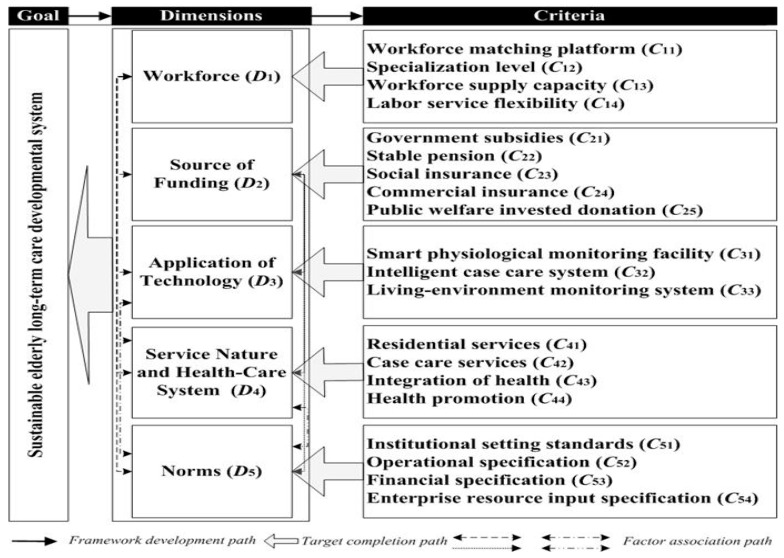
Evaluation framework for sustainable ageing health care in communities.

The first step in establishing an evaluative framework is to determine the final goal of this study; however, to achieve these sub-goals, the basic components must be defined, and sub-goals must be constructed from the key criteria identified in other studies. These elements are then organized into a framework that outlines the overall system standards for the objectives, aspects, and sub-goals to enhance the capacity for developing sustainable long-term ageing health care (Figure 1).

As indicated in Figure 1, this study aims to establish a framework that can provide comprehensive indicators for assessing the sustainability of long-term elderly-care systems. Norms were considered in parallel to the studies of workforce, source of funding, the application of technology, service nature, and health-care systems, due to the general recognition of being independent of one another. The “norms” of the research and the evaluation system is the fifth dimension. When considering the sustainability of a long-term ageing health care system, failing to recognize the normative dimension may result in overlooking valuable discussions of potential problems. The criteria listed in Figure 1 were extracted from the discussion in the previous two sections, and the sub-goals were achieved in five dimensions. All criteria for mining extraction in this section require identification. In the following sections, predictive testing is used to determine each evaluation criteria, and then the final study questionnaire is evaluated.

### 2.2. Pre-Test: Examination of the Indicator Framework

The main purpose of this chapter is to evaluate the reliability of the evaluation framework; therefore, the operation described in this section is divided into two phases. The first stage is to test the integrity of the overall framework of indicators, and the second stage is to test the importance of the indicator system. As there was no clear indicator framework in the past, it is very important to test the integrity of the overall framework. Therefore, this research adopted the semi-structured questionnaire design method combined with expert interviews. At this stage, the experts would add or delete the dimensions and criteria of the assessment framework according to their own professional knowledge and experience, and revise the names and definitions of the criteria. The second phase is devoted to testing the guidelines. This stage adopts the structured questionnaire design method and uses experts as the respondents of the questionnaire. The questionnaire scale ranges from 0 to 10, where 0 represents “extremely important”, 10 represents “very important”, and 5 represents “normal”. If the score is less than 5, the importance of the criterion is not enough and should be deleted, while a score of 7.5 or higher indicates the criterion is important and should be accepted. Therefore, criteria with a score between 5 and 7.5 would be discussed with experts after completion of the next round of investigation, until the overall guidelines achieve convergence (Table 1).

The survey time for the pre-test was 1 June to 23 July 2017, and a total of seven experts were investigated. Among them, three practical experts, all of whom had worked in the public sector for long-term elderly care policy development, were currently operators of long-term ageing health care in institutions, and most of them were currently consultants for long-term ageing health care policies. The other three experts were research scholars in the field of long-term ageing health care. In the first phase, the experts did not expand or simplify the framework of the guidelines, nor did they modify the names and definitions of the guidelines. During the first round of the second phase, 14 of the 20 criteria were above the acceptance threshold of 7.5, six were between 6–7.5, and no criteria were below the deletion threshold of 5. Of the six criteria between 6 and 7.5, five were between 7 and 7.5: labor service flexibility (*C*_14_), which was 7.2; government subsidies (*C*_21_), which was 7.4; stable pension (*C*_22_), which was 7.4; commercial insurance (*C*_24_), which was 7.3; and living-environment monitoring system (*C*_33_), which was 7.3. Enterprise resource input specification (*C*_25_) had a value of 6. Therefore, a second round of opinion surveys was conducted for these six criteria, among which the experts believed that the five criteria between 7 and 7.5 were important factors for elderly long-term care systems and recommended retaining them. The two experts giving lower scores further explained their evaluation criteria. Regarding the enterprise resource input specification, the experts believed that it is a manifestation of corporate social responsibility. While support for the introduction of enterprises would help the sustainable development of long-term care systems for the elderly, the experts also stated that enterprises can expand their business plans by giving donations to non-profit institutions; however, such plans must be carefully considered. Therefore, while the two experts raised strong doubts and gave lower scores, this explained the way in which the criteria were judged by the experts. According to the experts, when the importance of the identification of an item is judged, if the item is regarded as important, it has a higher score. Therefore, the guidelines for the evaluation framework for this study were retained. Overall, after the two-stage revision of the indicator system, the experts believed that all indicators are due to this reservation. Therefore, this study uses this set of indicator systems for subsequent research.

### 2.3. The Research Process of Developing a Sustainable Improvement Strategy

In the MCDM field, the DANP-mV model is a hybrid method consisting of two technologies: DANP (DEMATEL-base on ANP) and the Modified VIKOR technique [9,45]. The primary purpose of this approach is to propose a fundamental improvement strategy for alternatives in the system perspective to achieve the desired level of the original project objectives; thus, we must first understand the gap between the satisfaction of each criterion and the aspiration-level (gap in abbreviation), and then, we can evaluate each alternative by comparing the weighted gaps [46]. After determining the worst performing criteria, we can understand the influential relationship of the entire system through the influential network relation map (INRM), and then we can suggest fundamental improvement proposals for the alternative [46].

In the past, many scholars applied this method to different fields and proposed a systematic and fundamental improvement strategy for the topic; such as scholars in China, who put forward suggestions for improvement in the public open space of Harbin University [47,48]. They stated that although the waterbody has an important impact on increasing the satisfaction of the public development space, the natural conditions of Harbin would cause the waterbody to have unsustainable characteristics. Therefore, it was recommended to consider it from a system perspective, in order to improve public satisfaction with the public development space by improving plant configuration and density. Another example is the article on the sustainability of Taiwan’s Cultural and Creative Industries Park [49]. The main problem facing Taiwan’s Taichung Cultural and Creative Industry Park (TTCIP) is the lack of economic vitality. While the park continues to invite creative communities to host many events to attract consumers/visitors, it is not very effective. In the case of limited resources, is it necessary to invest resources in this way? This paper discusses the direction of improvement from the perspective of the mutual influence among various standards, and points out that the ability to activate the economy should include high-quality information transmission. Therefore, this paper argues that the operation of administrative organizations should be more efficient, and that it is necessary to propose improvement suggestions from a systemic perspective [48,49].

**Figure 2:**
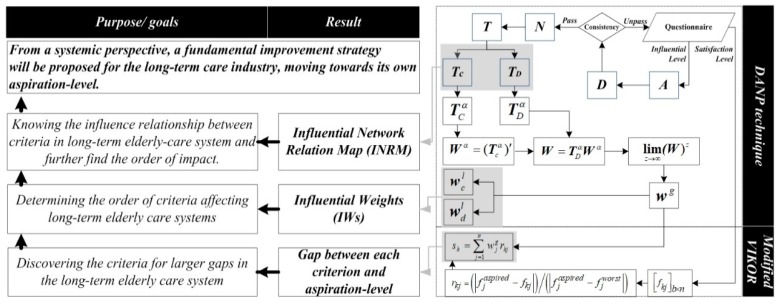
Operation and purpose of the proposed method.

The development of the long-term care industry for the elderly must be considered in terms of labor, funding sources, technology applications, the service nature, and the health care system, as there is a high degree of interrelationship between these four dimensions [50,51,52]. Therefore, the sustainability of the long-term ageing health care industry must be viewed from a systemic perspective [53]. In order to achieve sustainability of the long-term ageing health care system, we must first measure the poorly performing criteria. This part of the calculation integrates the influential weights (IWs) and gaps of each criterion, and then further looks for the criteria with the largest gap. After identifying the poorly performing criteria, it is necessary systematically to propose improvement strategies. This part uses INRM to understand which factors affect the criteria with the largest gap. Then, under this influence relationship, an improvement strategy is proposed. Among them, INRM and IWs are the results of the DANP technique, and the gap of each criterion is the result of the Modified VIKOR technique. The operation and purpose of the overall method are shown in Figure 2. For detailed steps, please refer to Appendix A.

## 3. Empirical Case: Analysis of Influential Degree in Taiwan’s Long-Term Ageing Health-Care System

This section first introduces the current situation of the long-term aging health-care system in Taiwan, and then determines the problems through a survey regarding satisfaction degree. Finally, it proposes a systematic improvement method to improve the case. Therefore, this section is divided into three parts: a background description of Taiwan’s long-term care system; data collection, analysis, and results display; and suggestions and improvement strategies.

### 3.1. Background Description of Taiwan’s Long-Term Ageing-Care System

In 1993, Taiwan’s population structure began to enter an ageing state. More than 14% of the population was older than 65 in 2018, and the birth rate moved to a negative number [54]. In this population structure change, such as the increase in demand for elderly care services, falling supplies, and other related issues have emerged consecutively. Taiwan’s government also began to study this problem, and in 2000, it formed a long-term care team. After eight years of research, this team proposed a long-term care (LTC) program (known as Long-term Care 1.0 in Taiwan), which covers disabled people, elderly people over 65 years old, mountain natives aged 55 to 64, individuals aged 50 to 64 years old who practice IADLs (instrumental activities of daily living), and individuals who are disabled and live alone [55].

Among them, the service items include care service, wheezing service, home care, rehabilitation service, auxiliary aid, shuttle service, food delivery service, and long-term institution check-in service. Ten years after the implementation of the plan, Taiwan enacted the Long-Term Care Service Act in 2017 (known as Long-term Care 2.0 in Taiwan), which expanded the scope of services to include preventive care and created backward connections to daily care and peace of mind for the end of life in the home. The purpose of Long-term Care Service 2.0 is to reduce the burden on caregivers and improve the quality of life and dignity of the elderly. In addition, it provides service bases with different levels, scales, and functions, as well as related services, such as small-scale multi-functional care units, dementia care, caregiver service bases, community preventive care, integration of indigenous communities, prevention of disabilities, delay in disabilities, preparation at discharge, referral care after discharge, home care, etc. To put it simply, Long-term Care Service 2.0 is based on the “prevention-medical-care” system [56].

Long-term care services are extensive and complex, thus, Taiwan’s care system is divided into three levels (A, B, C), each with a different role. Class C, known as the Hutong Long-Term Care Station, is the lowest level unit, which mainly provides a variety of care functions, such as short-term daily care in the village office or service center or meals for care recipients. Class B is known as the Multi-Function Long-Term Health Centre. Its main role is to achieve preventive health care through the integration of various functional departments; for example, after the patient is discharged from the hospital, the hospital sends the patient information data to a Class B care center for subsequent home care. The B-level care center conducts follow-up care needs assessment and sends the relevant information to the C-level care center. The Class C care centers provide services. Level A refers to the community integrated long-term medical service center. The main goal is to plan, develop, and promote the effective implementation of policies through resource allocation [57].

This care system further expands the coverage for dementia patients, as each county and city add a new care platform for dementia into the original disabled care center. The following explains the actual situation in more detail according to this index system.

● Workforce matching platform (*C*_11_)

The workforce matching platform can help the elderly by providing considerable assistance, sustain the supply of a long-term care labor force, and facilitate Taiwan’s employment channel platform matchmaking. Different levels of service centers for the elderly need different kinds of help, and the functions of each are not identical. The service centers for different types of professionals also provide related jobs [58].

● Specialization level (*C*_12_)

At a professional level, Taiwan demonstrates the need for care services to be assessed first by government professionals who can develop care plans and link long-term care resources. For those in need of medical care, if they cannot seek medical treatment due to disability, they would receive medical care from home nurses after being visited by medical evaluators. For those in need of rehabilitation, a physical or functional therapist would be sent to the patients’ home for rehabilitation services after diagnosis by specialists [59].

● Workforce supply capacity (*C*_13_)

At present, 45 colleges and universities in Taiwan have established long-term care-related departments, which can train about 5000 nursing professionals every year. Currently, there are about 140,000 licensed caretakers in Taiwan; however, only about 30,000 people are engaged in this industry. When the 2017 long-term care service 2.0 was launched, the shortage of care laborers was about 13,000 people [50]. In order to make up for this gap, the government recruited the required labor force from fresh nursing graduates, and encouraged middle-aged people and graduates of other majors to enter the long-term care industry.

● Labor service flexibility (*C*_14_)

The average monthly salary of the working population in Taiwan is about USD $1436, while the average monthly salary of long-term care personnel is only about USD $734 to USD $834. It can be found that salaries in the long-term care industry are lower than those of other industries [57]. In the past, most long-term care service providers paid workers’ compensation and used the “uniform package”, which was calculated in time segments. In order to utilize labor more effectively, the “item-by-item pricing” approach is currently used. The purpose of support is to provide more flexible work time compensation, enabling employees to perform their duties more effectively.

● Government subsidies (*C*_21_)

At present, the financial resources of the long-term care system in Taiwan are mainly obtained through increasing taxes, and subsidies are based on the two criteria of losing self-care ability and economic status. The financial subsidy in 2017 was set at 100% for individuals earning USD$343 to USD $515 per person per month, 90% for individuals earning USD$515 to USD $740 per person per month, and 70% for general subsidized care. For remote areas, due to the need for medical care or long-term care services, transportation assistance and caregivers are needed; hence, it is necessary to subsidize transportation and other expenses for severely disabled people in disadvantaged groups, or to rent medical equipment for family health care, such as the home medical equipment needed for rehabilitation and subsidies to improve barrier-free spaces inside and outside the home, as well as elderly care allowances that qualify for long-term care needs [54]. In the past, the financial resources during long-term care plan 1.0 were based on commercial insurance, and such stable financial resources had significant impact on the sustainability of the long-term care system. Therefore, there is still much discussion in Taiwan regarding the transfer of long-term care services from commercial insurance to taxation.

● Stable pension (*C*_22_)

Retirement pensions are the important basis of giving the elderly an independent life. After the Second World War, the global economy shifted from a recession to a period of high growth [58], thus, Taiwan’s aged population experienced a period of rapid growth, and in this context, the older generation gained a better concept of savings. The the issue of the elderly population is of increasing concern for young people; however, elderly pension is stable, which has a certain stabilizing effect on society and families [60]. In Taiwan, a pension is a labor protection mechanism, as the government requires employers to pay a certain pension insurance tax every month, as based on the wages of the workers. (Employees can also increase the amount of insurance according to their own wishes.) When workers retire, they can receive a pension from the government and their own employee insurance, as stored in the manner prescribed by law.

● Social insurance (*C*_23_)

The national health insurance system is a social medical insurance system [61], which all Taiwanese can enjoy together; however, this system only solves the medical part of the long-term care system. The subsequent long-term ageing health care is currently dependent on other systems for connection [62].

● Commercial insurance (*C*_24_)

Commercial insurance is used to make up for the deficiency of the public insurance system [63]. Therefore, different purchasing behaviors are generated according to individual needs, incomes, and characteristics [64]. This is also true in different areas of Taiwan, such as northern Taiwan, where there are more and higher purchases of commercial insurance, as it is the political and economic core of Taiwan.

● Public welfare invested donation (*C_25_*)

Encourage relevant corporate philanthropy, promote corporate social responsibility practices, and achieve the integration of corporate and public welfare undertakings in corporate donations [31].

● Smart physiological monitoring devices (*C*_31_)

Smart physiological monitoring devices can measure the physiological state of the elderly at any time, and then transmit the data to a remote database for interpretation [35]. At present, the main smart devices in Taiwan can be divided into two categories; one is specialized devices, such as smart mattresses, while other is consumptive devices, such as smart bracelets, crutches, and shoes. Specialized devices are mostly used in large medical and long-term care institutions as they are more expensive [65].

● Intelligent case care systems (*C*_32_)

At present, some large medical care companies in Taiwan are using cloud ERP systems as a management platform in medical care to manage individual cases. This management platform records the past treatment process, rehabilitation, and related follow-up services, in order to enable the medical long-term care agency to make more efficient use of resources and maximize the benefits [66].

● Living-environment monitoring systems (*C*_33_)

Living-environment monitoring systems mainly focus on the long-term care system modularization of merchandise. With living-environment monitoring, the emergency medical system can move quickly during a medical rescue [67]. Taiwan’s electronic communications technology industry is an interdisciplinary medical and long-term care services industry, and many companies have developed relevant integrated products.

● Residential services (*C*_41_)

At present, residential services mainly provide relevant services on the basis of the care management center; they provide timely housework and daily care, help with the provision of meals for caregivers, and body care services [68]. Care Management Central also provides a meal delivery service for elderly people living alone. Upon assessment, the center provides this service to elderly people who are unable to prepare their own meals [69].

● Case care services (*C*_42_)

Taiwan is promoting a community-based ageing mechanism to provide diversified and differentiated services through the long-term ageing health care service system of caring communities [55].

● Integration of health (*C*_43_)

Since the ultimate goal of a health care system is to provide an integrated preventative care service to those in need, effective integration of the personal home environment, medical system, long-term ageing health care system, and government-related units must be carried out [6,37]. Currently, under the Long-Term Care Service 2.0 policy in Taiwan, different functional units have been integrated, and the health care system has been divided into three levels (ABC), which have different responsibilities.

● Health promotion (*C*_44_)

Health promotion is currently developing in Taiwan in the two main directions of prevention and the delay of disability. The government also encourages related medical consortiums or school units to propose relevant courses, such as using physical fitness training to prevent the degeneration of functions or delaying the speed of dementia through the stimulation of sound waves [40].

● Institutional setting standards (*C*_51_)

At present, Taiwan’s long-term Care Services Act regulates the management of legally registered long-term care institutions. Under this law, relevant regulations have been established [56].

● Operational specifications (*C*_52_)

The Long-Term Care Services Act applies to legitimate long-term care organizations, including institutional layout, business categories, external advertising, service content and cost criteria, staffing, referral services, and links between healthcare and long-term ageing health care systems. The organization’s safety management, service quality standards, supervision by competent government departments, and illegal penalties are clearly stipulated [70].

● Financial specifications (*C*_53_)

The financial regulations for long-term ageing health care organizations mainly reveal the process of operating performance management with the principle of financial transparency [71], and there are relevant explicit regulations in the Long-Term Care Services Act.

● Enterprise resource input specifications (*C*_54_)

In order to promote the development and subsidies of long-term care system-related resources, the sources of relevant funds have been specified in the Long-Term Care Services Act, among which donation income is one source of funding [56,71]. However, monetary donations must comply with the provisions of income tax law regarding donations to charities. In Taiwan, charitable organizations have different norms, as based on individual and for-profit institutions. Individuals can donate to legally-registered charities, with the total donation not exceeding 20% of an individual’s personal income in the current year. The donations made by profit-making institutions shall not exceed 10% of the revenue in the current year. However, there is no restriction if the donation is approved by the Ministry of Finance.

### 3.2. Data Collection

This study used a questionnaire survey to collect the data. The first interview phase was with experts from the industry and academic circles. Industry officials and academic representatives are different types of stakeholders and system participants, and these different types of experts often represent insights from different perspectives. However, because the officials are in charge of the implementation and promotion of the policy, there could be problems in the investigation or avoidance of the actual situation. In order to avoid the impact of such bias, this study excluded one official. The academic experts were those who were actually involved in business management and academic study in theoretical development and practice. The second interview phase was related to the use of long-term elderly care systems. The respondents were asked to evaluate 20 criteria in the framework, as based on their perception of the long-term elderly care system. Among the respondents, the difference between the experts and users was that experts often used a comprehensive and futuristic perspective to make evaluations. The use of relevant individuals has the characteristics of locality, and the selections are usually based on respondents’ own feelings. Therefore, this study simultaneously conducted evaluation from the two groups of the experts and the public.

In the first stage, a total of 10 questionnaires were distributed using the face-to-face distribution method. In addition to completing the structured questionnaires, the experts were interviewed to collect their opinions. The interviewed experts all expressed their willingness to accept surveys and interviews; therefore, all 10 questionnaires were valid. Among the experts, three scholars were engaged in relevant academic research and teach in a university. More importantly, they had led or participated in community-based long-term health care related research and planning. The other seven industry experts were professionals in health management or management of the long-term care industry with at least 10 years of related work experience. Although three of the industry experts were currently business operators, they were previously consultants for the government’s long-term ageing health care policy, and one of them was once the head of a government-related business unit. The other four industry experts were operators of long-term ageing health care businesses. A total of 10 questionnaires were distributed to the second-stage expert respondents. The respondents were the same as the first-stage experts, and the same face-to-face interviews were conducted; therefore, all 10 questionnaires were valid. A total of 330 questionnaires were distributed to relevant users. As some questionnaires had missing values, 15 questionnaires were considered invalid; therefore, the total number of effective questionnaires was 315. The respondents were from the northern, central, southern, and eastern regions of Taiwan and the outlying islands. They have different levels of education, different occupations, and different monthly incomes. The data were collected between 1 August 2017 and 1 February 2018. The expert interviews took place between 1 August and 23 October 2017, and the average time required for each questionnaire was 2.5 to 3 hours. The public survey part took place from 1 October 2017 to 1 February 2018.

### 3.3. Results and Discussion

**Figure 3:**
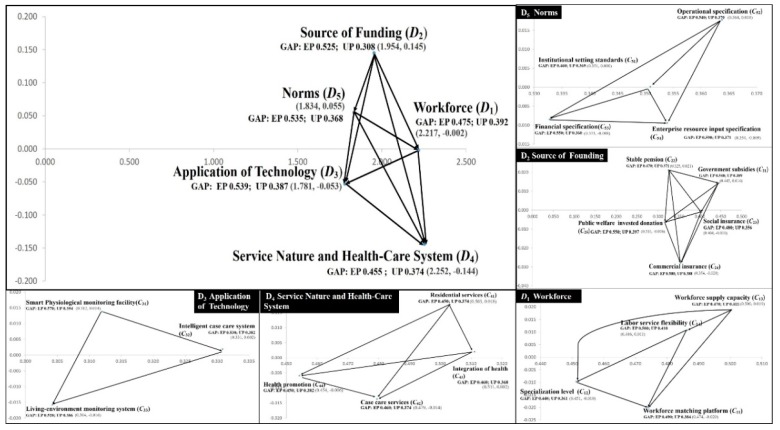
Influential network relation map (INRM) of the total influence relationships.

This study constructed an index system for long-term ageing health care, which contains five dimensions and 20 criteria. The interactions of the various factors among the systems were investigated and identified according to the INRM of the DANP technique, as shown in Figure 3 (For detailed calculation results, please refer to Appendix B, Table A1, Table A2, Table A3, Table A4, Table A5, Table A6 and Table A7). The vertical axis of INRM represents the level of influence, where a higher level of influence indicates the factor’s greater impact within the overall system; that is, if this factor changes, other factors would be affected. Therefore, when policy makers need to formulate improvement strategies, there are more effective ways to conceive factors based on the source of influence, especially in the case of limited resources. According to Figure 3, the order of influence is: source of funding (*D*_2_), norms (*D*_5_), workforce (*D*_1_), application of technology (*D*_3_), and service nature and health-care system (*D*_4_).

These results indicate that source of funding (*D*_2_) is the foundation of the long-term ageing health care system, and policymakers should base their plans according to funding, and then, observe the structure to address the other four aspects. The impact of this strategic development, which is referred to as the Coping Measures and Root Cause Improvement Strategy in this study, is a more effective and efficient approach. The use of this improved strategy can promote the sustainability of the long-term health care system.

After understanding the source and impact order of each dimension, the parts of the criteria in each dimension were observed. First, the factor with the most impact on the source of funding (*D*_2_) dimension is stable pension (*C*_22_). The order of influence is stable pension (*C*_22_), government subsidies (*C*_21_), social insurance (*C*_23_), public welfare investment donation (*C*_25_), and commercial insurance (*C*_24_). The factor for influencing the source in norms (*D*_5_) is operational specifications (*C*_52_), and the order of influence is operational specifications (*C*_52_), institutional setting standards (*C*_51_), financial specifications (*C*_53_), and enterprise resource input specifications (*C*_54_). The third dimension is workforce (*D*_1_), and the factor for influencing the source in this dimension is workforce supply capacity (*C*_13_). The order of influence is: workforce supply capacity (*C*_13_), labour service flexibility (*C*_14_), specialization level (*C*_12_), and workforce matching platform (*C*_11_). However, the smart physiological monitoring device (*C*_31_) is the source of influence of the application of technology (*D*_3_), and the order of influence is: smart physiological monitoring devices (*C*_31_), intelligent case care system (*C*_32_), and living-environment monitoring system (*C*_33_). Finally, residential services (*C*_41_) is the source of influence for service nature and the health-care system (*D*_4_), and the order of influence is: residential services (*C*_41_), integration of health (*C*_43_), health promotion (*C*_44_), and case care services (*C*_42_).

After understanding how to develop a complementary and rooted improvement strategy, the next step was to determine the biggest difference from the ideal state, which this study calls the biggest gap. The biggest gap can be found using the modified VIKOR technique, and the results of the operation are shown in Table 2. Since the long-term health care system has been implemented in Taiwan for some time, this study explored long-term health care from the user perspective (user performance; UP), as well as from the perspectives of the Policy makers and implementers (expert performance; EP). Two different perspectives were used to explore the problem criteria for long-term ageing health care systems.

First, according to the total gap value in Table 2, the value of UP is 0.380, which is better than the EP value of 0.502. These results represent different degrees of perception between the users (people), policy makers, and implementers (experts). However, both sides believed that there was room for improvement in the system, and that it required improvement. This study then observed the gap value of each dimension. The experts believed that the dimension of the largest gap is Application of Technology (*D*_3_). The order of the gap from large to small is *D*_3_ (0.539), *D*_5_ (0.535), *D*_2_ (0.525), *D*_1_ (0.475), and *D*_4_ (0.455). The public believed that the dimension with the largest gap is Workforce (*D*_1_). The order of gap from large to small is *D*_1_ (0.392), *D*_3_ (0.387), *D*_2_ (0.380), *D*_4_ (0.374), and *D*_5_ (0.368).

The results indicate that the level of experience of the experts and the public only presents a partial consensus, such as the ordering of *D*_3_, *D*_2_, *D*_4,_ *D*_3_, and *D*_5_. The process of filling out the questionnaire found that such cognitive gaps mainly came from differences in opinions. Experts are usually policy designers or industry executives; therefore, they adopt the perspective of design or performance. However, people have strong feelings about the implementation of policy, and this feeling is often affected by the relationships among the media, relatives, friends, and their own impressions. As some of the topics in this study, such as regulations or technology products, are related to professional thinking, and because the respondents were mainly seniors over the age of 65, there might be some degree of error in the perception of this topic. In order to avoid such biases, although the public’s point of view was valued, this study used expert satisfaction in the formulation of support and root improvement strategies.

According to the sorting of the dimension gap, the first description is the application of technology (*D*_3_). In this dimension, the criterion of the maximum gap (Biggest gap) is smart physiological monitoring devices (*C*_31_), which is 0.570. The order is *C*_31_, *C*_32_, and *C*_33_, followed by norms (*D*_5_) in this dimension, and the criterion for the maximum gap is enterprise resource input specifications (*C*_54_), which is 0.590, followed by *C*_53_*, C*_52_, and *C*_51_. This is also the biggest gap of all criteria. The following dimension is the source of funding (*D*_2_). In this dimension, the largest gap criterion is commercial insurance (*C*_24_) with a value of 0.580, followed by *C*_25_, *C*_21_, *C*_23_, and *C*_22_. The next dimension is workforce (*D*_1_). In this dimension, the maximum gap criterion is Labour service flexibility (*C*_14_), which is 0.500, followed by *C*_11_, *C*_13_, and *C*_12_. The last dimension is service nature and health-care system (*D*_4_). In this dimension, the maximum gap criteria are case care services (*C*_42_) and integration of health (*C*_43_), both of which are 0.460, while secondary influences are *C*_41_ and *C*_44_ (Table 2).

The principle of smart physiological monitoring devices (*C*_31_), which was the biggest gap in the application of technology (*D*_3_), is that large home monitoring systems or professional medical care monitoring systems cannot be extended to every consumer; therefore, current intelligent monitoring systems mainly consist of consumer electronic products, as launched by manufacturers. However, this can lead to incompatible monitoring systems and create gaps in care services. As a result, the intelligent monitoring systems criterion was the source of the application of a technology dimension. Therefore, to improve the application of technology dimension, this study suggested improving the precision of consumer electronics using the data of medical units, integrating the information of consumer electronics with the medical monitoring of large hospital systems, and allowing data to flow instantly in two directions.

Regarding Norms (*D*_5_), the factor of gap’s biggest problem is Enterprise resource input specifications (*C*_54_), which is due to Taiwan’s current regulations restricting the direct investment of profit-making institutions in long-term ageing health care systems. During the interviews, many experts emphasized that the participation and resource investment of profit-making institutions are important bases for the stable development of long-term health care systems for the elderly. However, the government remains concerned that if laws and regulations are loosened, the profit-making characteristics of profit-making institutions might lead to essential changes for non-profit institutions. In this dimension, the source of impact is Operational Specifications (*C*_52_). Currently, the government’s operating regulations are designed to prevent misconduct in operations, rather than sustainable development. Therefore, the idea of adding a transparent navigation drainage mechanism to replace restrictions in the operational specifications could allow for-profit companies to effectively and efficiently channel resources into this career.

In the Source of Funding (*D*_2_) dimension, the biggest gap problem criterion was Commercial insurance (*C*_24_). The current low purchase rate of commercial health insurance in Taiwan is due to the limitations of salary levels and a lack of sufficient public awareness. While there is a robust health care system in Taiwan, the elderly tend to ignore care and maintenance after receiving medical care, which is a large future expense in their retired life. In addition, young people generally face the problem of insufficient wages, which leads to a lack of willingness to purchase commercial health insurance. Such issues would lead to an increase in social welfare insurance expenditures, thereby affecting the sustainable development of the long-term ageing health care system.

If only relying on individual purchases of commercial health insurance, the dilemma of the problem of insufficient salaries remains, and the funding source of the long-term ageing health care system is still limited. “Funding” should not be regarded as a negative cause. Although the amount of funding positively correlates with the long-term development of the system, “exploring more sources” is not the only point of attention, as “effective use of funding” can also ensure that funding is well allocated. Reducing the waste of expenses could allow assets to be effectively utilized and reach those who need help; for example, the purchase of some commercial insurance considers the price sensitivity of consumers [72], and also allows doctors to play the role of a bridge between insurance clients and insurance practitioners. Those in need of insurance can communicate with insurance practitioners through professional advice, and then, find the type of insurance that suits them best. This will not only avoid consumer price cost sensitivity issues, it will also mean that consumers can buy business insurance that meets their needs, and thus, effectively reduce their risk of financial bankruptcy. Finally, the system’s sustainability can help more people in need.

For the long-term ageing health care system to achieve sustainability, the performance of funding sources must be improved. A pension is a very important influencing factor, because it is the only source of income for most elderly people. If seniors’ pensions are in a stable state, they would be able and willing to purchase appropriate commercial health insurance, which can serve as an additional source of funding for the long-term ageing health care system and effectively reduce the dependence on social welfare insurance. As a result, more sustainable development of the long-term health care system for the elderly can be provided. Commercial insurance providers can offer appropriate commercial insurance, thereby providing additional attention to the funding sources of the long-term ageing health care system, reducing dependence on social welfare insurance, and enabling the development of the elderly long-term health care system into a more sustainable state.

Regarding the workforce (*D*_1_) dimension, the biggest gap problem is Labour service flexibility (*C*_14_). Taiwan’s remuneration for care services is a system of unified parcel time; that is, time is used as the pricing standard for long-term ageing health care services. Regardless of the number of care services that a caregiver is required to perform within a given time period, only a fixed salary is paid to the caregiver. In other words, the number of service items is not associated with the compensation. However, in practice, a caregiver often needs to increase or decrease the service time according to his/her abilities and performance. If the number of work items is different, but the same salary is received, it would result in shortage of labour. Therefore, considering the ability of the caregiver and providing reasonable compensation could enable the workforce in the long-term ageing health care system to be effectively promoted.

Regarding the Service nature and health-care system (*D*_4_) dimension, the biggest gap problem is case care services (*C*_42_) and integration of health (*C*_43_). The current long-term care case manager and home care worker system in Taiwan is the relevant system implemented by the government to implement case management services. The main services are based on hospital discharge preparation, home visits, and follow-up care services. The purpose of this system is to enable service users to receive proactive care. The reason for the largest gap is the immediacy of demand from the service demanders. Since service demanders have more urgent need characteristics, case managers must make considerable clarifications in accordance with certain regulations and procedures when handling follow-up care. Such a process is less likely to respond to the needs of service demanders in real time. Residential services are the root cause of all impacts in this dimension. Currently, the service items offered by home caregivers are mainly planned and assigned by the case manager after judging the needs of individual cases. However, the number of service applicants continues to increase year by year; therefore, case managers are taking longer to respond to the needs of real users. Home care workers are often front-line service personnel, and are most familiar with the needs of the patients. In the past, the home care workers only engaged in factional work, as based on the assessment results of case managers; therefore, it is suggested that the services provided by home care workers should be flexible enough to meet the needs of users in real situations.

With regard to the integration of Taiwan’s long-term care system, the preparatory office of the government authority was established in September 2018, which is responsible for the integration of the long-term health care system in Taiwan; however, in the past, its related units and organizations adopted different systems, and efforts have been made to integrate and test the new system. In the past, the integration of health care systems was mostly based on consideration of the part of the system, and was less concerned with case requirements; therefore, it is recommended that home managers provide immediate feedback, which can be integrated into the health care system and used to adjust relevant structures. In general, because home care workers are the front-line service unit, they are also the unit that can identify problems the easiest; therefore, it is recommended to keep home care workers’ job items flexible, and design a mechanism that integrates the views of the immediate response service provider with the needs of the demander, in order to provide a basis for the sustainable development of the elderly health care system.

One aspect of the largest gap in the four areas is application technology (*D*_3_). This problem is mainly due to the difference between the precision of physiological and home-monitoring data and medical equipment. The physiological monitoring system is a type of consumer electronic product. A home-monitoring system has two characteristics; the first is the expansion of the physiological monitoring system, which is also a consumer electronics product; the second is the monitoring of the home environment. Such products are usually communication products. While these two types of products offer the advantage of immediate and low-cost monitoring and development, they also have lower precision than medical devices. However, for medical behaviour, high complexity is a necessary choice. Therefore, a problem that must be considered is how to improve the sophistication of consumer electronics and telecom products to integrate real-time monitoring health data with medical systems. To overcome this problem, it is necessary to ensure that multiple sources obtain sufficient funds, formulate appropriate policies and regulations, and protect the privacy of the health data model to establish certain basic standards for integration. Finally, it is important to have a high-quality workforce that is familiar with high quality medical maintenance applications and the implementation of related technology.

In summary, this study found that the two criteria of stable pension (*C*_22_) and government subsidies (*C*_21_) are the key to the government’s sustainable development of long-term ageing health care systems. The government must effectively adjust and control these two items. If a senior’s pension is in a stable state and there is a certain degree of government subsidy, the government can maintain the transcendence of non-profit businesses without the need specifically to introduce resources from profit-making business units. However, when the personal pensions of the elderly are in an unstable state, and government subsidies are relatively scarce, it is necessary to appropriately introduce resources from profit-making business units. According to the government’s current budget on subsidies for the long-term aging health care system and the current self-sustaining savings rate of the elderly, it is necessary to further conceive relevant laws and regulations, in order to maximize the benefits of resources, provide long-term ageing health care for the elderly, and achieve sustainable development.

## 4. Conclusions

Long-term ageing health care systems must be designed from a comprehensive perspective. Therefore, the purpose of this study is to determine the core control factors, to understand the current issues of the system through the influence relationship, and then, propose a set of sustainable long-term ageing health care systems. There are three findings in this study. The first is that this study defines the framework of a long-term ageing health care system into five dimensions and 20 criteria. The second is that if the long-term aging health care system is to achieve sustainable development, a stable pension (*C*_22_) and government subsidies (*C*_21_) must be given priority. The last point is that, the main problem of the unsustainable long-term ageing health care system in Taiwan lies in the enterprise resource input specification, as stable pension (*C*_22_) and government subsidies (*C*_21_) are the source of influence. Therefore, this study aimed to improve the needs of the long-term health care system to be in accordance with government subsidies for the elderly long-term ageing health care system, including the budget for personal use and elderly current savings rate, and then further designed and formulated loosened and relevant laws and regulations, as well as other matters, in order that resource benefits are maximized to allow the long-term ageing health care system to achieve the effect of sustainable development.

This study has two limitations. First, it proposed an index framework that can be used as a reference for other research; however, because local areas have a certain degree of heterogeneity, future research should further explore regional characteristics when adopting this index framework. Second, this study used the DANP-mV model, which belongs to the performance evaluation method of the additive type, while the practice of performance evaluation problems often belongs to the condition of non-addition. Therefore, further research can consider using the non-addition method for the evaluation of overall performance.

## Figures and Tables

**Table 1:** Evaluation framework long-term ageing health care in communities.

Dimensions	Criteria	Descriptions	Cited Reference
Workforce (*D*_1_)	Workforce matching platform (*C*_11_)	Services and programs based on labour providers can match those with care demands.	[20,21]
	Specialization level (*C*_12_)	According to the provision of long-term ageing health care service personnel, there are qualified professional licenses.	[4,20]
	Workforce supply capacity (*C*_13_)	Assess the adequacy of the manpower available to provide long-term ageing health care services.	[22]
	Labour serviceable flexibility (*C*_14_)	Evaluate the total number of service hours required to provide services to the supply side based on the provision of health care services.	[20,23]
Source of Funding (*D_2_*)	Government subsidies (*C_21_*)	Government subsidies for community long-term health care services.	[14,24,25]
	Stable pension (*C_22_*)	Retirement system, the level of pensions, and the degree of stability.	[26,27]
	Social insurance (*C_23_*)	Social health insurance can cover the extent of community long-term health care programs.	[17,24,28,29]
	Commercial insurance (*C*_24_)	According to the payment amount of long-term health care commercial insurance, the requirement of commercial insurance is selected by the elderly.	[25]
	Public welfare invested donation (*C*_25_)	Provide the extent of resources invested in service to the community through community health care.	[31,32,33]
Technology Application (*D*_3_)	Smart physiological monitoring facility (*C*_31_)	Assess the convenience of collecting the physical condition of the elderly for analysis.	[15,34]
	Intelligent case care system (*C*_32_)	Assess the function of daily life activities and the improvement of the information system of elderly customer relationship management.	[10]
	Living environment monitoring system (*C*_33_)	Internet of Things applications in smart city will provide integrated management for the use of an intelligent care services system.	[15,35]
Service Nature and Healthcare system (*D*_4_)	Residential services (*C*_41_)	Provide cleaning, laundry, housekeeping, and accompaniment to complete daily activities.	[17]
	Case care services (*C*_42_)	Provide daily and night care services to improve the services.	[10,36]
	Integration of health (*C*_43_)	Health management is integrated with long-term health care and medical services to provide action health management implementation program.	[23,37]
	Health promotion (*C_44_*)	Community-based health promotion activities include the provision of multiple services, such as senior citizens’ meals, physical fitness, psychology, health education, and generational integration	[10,38,39]
Norms (*D_5_*)	Institutional setting standards (*C_51_*)	Provide cleaning, laundry, housekeeping, and accompaniment to complete daily activities.	[31,33]
	Operational specification (*C_52_*)	According to the applicability of the enterprise operation of modern management health care system regulations.	[41,42]
	Financial specification (*C_53_*)	The health care sources of funding and the level of transparency in the use and disbursement of government subsidies.	[43]
	Enterprise resource input specification (*C_54_*)	Integrate enterprise resources to inject the integrity of the tax code of community long-term health care services.	[42,44]

**Table 2:** Evaluation results of Taiwan’s long-term ageing health care system.

Dimensions/Criteria	Local Weight	Global Weight	Experts Performance	Gap	User Performance	Gap
**Workforce (*D*_1_)**	**0.221**		**5.246**	**0.475**	**6.081**	**0.392**
Workforce matching platform (*C*_11_)	0.257	0.057	5.100	0.490	6.159	0.384
Specialization level (*C*_12_)	0.241	0.053	5.600	0.440	6.385	0.361
Workforce supply capacity (*C*_13_)	0.252	0.056	5.300	0.470	5.889	0.411
Labour service flexibility (*C*_14_)	0.250	0.055	5.000	0.500	5.901	0.410
**Source of Funding (*D*_2_)**	**0.180**		**4.746**	**0.525**	**6.200**	**0.380**
Government subsidies (*C*_21_)	0.240	0.043	4.600	0.540	6.105	0.389
Stable pension (*C*_22_)	0.166	0.030	5.300	0.470	6.293	0.371
Social insurance (*C*_23_)	0.217	0.039	5.200	0.480	6.436	0.356
Commercial insurance (*C*_24_)	0.207	0.037	4.200	0.580	6.124	0.388
Public welfare invested donation (*C*_25_)	0.171	0.031	4.500	0.550	6.032	0.397
**Application of Technology (*D*_3_)**	**0.183**		**4.607**	**0.539**	**6.129**	**0.387**
Smart physiological monitoring facility (*C*_31_)	0.316	0.058	4.300	0.570	6.064	0.394
Intelligent case care system (*C*_32_)	0.351	0.064	4.700	0.530	6.175	0.382
Living-environment monitoring system (*C*_33_)	0.332	0.061	4.800	0.520	6.143	0.386
**Service Nature and Health-Care System (*D*_4_)**	**0.239**		**5.448**	**0.455**	**6.256**	**0.374**
Residential services (*C*_41_)	0.251	0.060	5.500	0.450	6.258	0.374
Case care services (*C*_42_)	0.254	0.061	5.400	0.460	6.258	0.374
Integration of health (*C*_43_)	0.262	0.062	5.400	0.460	6.315	0.368
Health promotion (*C*_44_)	0.234	0.056	5.500	0.450	6.185	0.382
**Norms (*D*_5_)**	**0.177**		**4.648**	**0.535**	**6.323**	**0.368**
Institutional setting standards (*C*_51_)	0.253	0.045	5.400	0.460	6.309	0.369
Operational specification (*C*_52_)	0.245	0.043	4.600	0.540	6.299	0.370
Financial specification (*C*_53_)	0.242	0.043	4.500	0.550	6.401	0.360
Enterprise resource input specification (*C*_54_)	0.260	0.046	4.100	0.590	6.287	0.371
**Total** **performance**			**4.982**		**6.196**	
**Total** **gap**				**0.502**		**0.380**

## Appendix A. Research Steps

The DANP-V model, which was adopted for this study, was proposed by Tzeng [46,47], who combined new concepts and techniques to solve complex and dynamic real-world problems.

*Step 1: Establish the direct influence relation matrix **A**.*

Data were obtained using the questionnaire, and the scales used in the questionnaire involved an integer score of 0, 1, 2, 3, or 4, ranging from 0 representing absolutely no influence to 4 representing very high influence, according to natural language criteria from linguistics. The respondent had to be an expert in the field and use the pairwise comparison method to evaluate the degree of influence of the criteria, and to show the degree to which each criterion *i* affects each criterion *j*. This matrix must be a *n* × *n* anonnegative matrix. The individual direct influence relation matrix ***A*** can be obtained by a survey.

(A1) $$A=\left[\begin{array}{ccccc} a_{11} & \cdots & a_{1j} & \cdots & a_{1n}\\ \vdots & & \vdots & & \vdots \\ a_{i1} & \cdots & a_{ij} & \cdots & a_{in}\\ \vdots & & \vdots & & \vdots \\ a_{n1} & \cdots & a_{nj} & \cdots & a_{nn} \end{array}\right]$$

*Step 2: Constitute the average direct influence relation matrix **D***.

When the number of experts surveyed is *H*, it means that there are *H* direct influence relation matrices. By averaging these matrix values, the direct influence relation matrix *D* of all experts can be obtained. The average scores of the *H* experts are $d_{ij}=\frac{1}{H}\sum_{h=1}^{H}a_{ij}^{}$. The average matrix is called the average direct influence relation matrix ***D*** and represents the degree of influence that one criterion exerts on another and the degree of influence that the criterion receives from another, as shown in Equation (A2).

(A2) $$D=\left[\begin{array}{ccccc} d_{11} & \cdots & d_{1j} & \cdots & d_{1n}\\ \vdots & & \vdots & & \vdots \\ d_{i1} & \cdots & d_{ij} & \cdots & d_{in}\\ \vdots & & \vdots & & \vdots \\ d_{n1} & \cdots & d_{nj} & \cdots & d_{nn} \end{array}\right]$$

*Step 3: Examine consensus.*

The value of consensus can be estimated by Equation (A3), which represents the level of experts’ consensus. The threshold of the average gap ratio is 5% in statistics; a value less than 5% implies a confidence level above 95%, which also represents a stable system. Conversely, if an unstable system is obtained, the first phase should be implemented again to verify whether data collection is correct and whether the number of experts is sufficient.

(A3) $$\mathrm{Average}\;\mathrm{gap}-\mathrm{ratio}\;\mathrm{in}\;\mathrm{consensus}(\%)=\frac{1}{n(n-1)}\sum_{i=1}^{n}\sum_{j=1}^{n}\left(\left|d_{ij}^{H}-d_{ij}^{H-1}\right|/d_{ij}^{H}\right)\times 100\%$$

*Step 4: Formulate the normalized average direct influence relation matrix*N.

The normalized average direct influence relation matrix N is acquired by normalizing the matrix ***D***. The matrix ***N*** is easily derived from Equations (A4) and (A5), in which all principal diagonal criteria are equal to 0:

(A4) N=b·D

(A5) $$b=\min\left\{\frac{1}{\max_{1\leq i\leq n}\sum_{j=1}^{n}d_{ij}},\frac{1}{\max_{1\leq j\leq n}\sum_{i=1}^{n}d_{ij}}\right\}$$

*Step 5: Construct the total influence relation matrix*T.

A continuous decrease of the indirect effects of problems moves with the powers of the matrix N, e.g., $N^{2},\ldots ,N^{\infty },$ and $\lim_{q\to \infty }N^{q}={\left[0\right]}_{n\times n},$ for $\lim_{q\to \infty }(I+N+N^{2}+\ldots +N^{q})=$ ${\left(I-N\right)}^{-1}$, where I is a n×n unit matrix. The total influence relation matrix ***T*** is a n×n matrix and is defined by $T={\left[t_{ij}\right]}_{n\times n},\;i,j=1,2,\ldots ,n$, as shown in Equation (A6).

(A6) $$T=N+N^{2}+\ldots +N^{q}=N(I+N+N^{2}+\ldots +N^{q-1})\;=N(I+N+N^{2}+\ldots +N^{q-1})(I-N){\left(I-N\right)}^{-1}\;=N{\left(I-N\right)}^{-1},\;\mathrm{when}\;\lim_{q\to \infty }N^{q}={\left[0\right]}_{n\times n}$$

*Step 6: Illustration of INRM*.

The total influence relation matrix T of INRM can be acquired using Equations (A7) and (A8) to generate each row sum and column sum in the matrix T, respectively.

(A7) $$o={\left(o_{i}\right)}_{n\times 1}={\left[\sum_{j=1}^{n}t_{ij}\right]}_{n\times 1}=(o_{1},\ldots ,o_{i},\ldots ,o_{n})$$

(A8) $$r={\left(r_{i}\right)}_{n\times 1}={(r_{j}{)}^{\prime }}_{1\times n}={\left[\sum_{i=1}^{n}t_{ij}\right]}^{\prime }{}_{1\times n}=(r_{1},\ldots ,r_{j},\ldots ,r_{n}{)}^{\prime }$$

where $o_{i}$ is the sum of a row in the total influence relation matrix T, which represents the total effects (both direct and indirect) of criterion/perspective *i* on all other criteria/perspectives ${\left[\sum_{j=1}^{n}t_{ij}\right]}_{n\times 1}$. Similarly, *r_j_* is the column sum in the total influence relation matrix T, which represents the total effects (both direct and indirect) of criterion/perspective *j* received from all other criteria/ perspectives ${\left[\sum_{i=1}^{n}t_{ij}\right]}^{\prime }{}_{1\times n}$. $\left(o_{i}+r_{i}\right)$ provides an index of the strength of the total influences given and received; that is, $\left(o_{i}+r_{i}\right)$ indicates the degree of importance of the criterion/perspective *i* in the system. In addition, $\left(o_{i}-r_{i}\right)$ provides an index of the degree of the cause of the total influence. If $\left(o_{i}-r_{i}\right)$ is positive, then criterion/perspective *i* is a net causer, and if $\left(o_{i}-r_{i}\right)$ is negative, then criterion/perspective *i* is a net receiver.

*Step 7: Construct the total influence relation matrix of criteria*$T_{C}$*and dimension*$T_{D}$.

The total influence relation matrix of criteria $T_{C}$ from each perspective (dimension or cluster), with different degrees of influence relation for the criteria, is shown in Equation (A9), where $\sum_{j=1}^{m}m_{j}=n$, m<n, and $T_{c}^{ij}$ as a $m_{i}\times m_{j}$ matrix.

(A9)

where $D_{m}$ is the *m*th cluster; $c_{mm}$ is the *m*th criterion in the *m*th dimension; and $T_{C}^{ij}$ is a submatrix of the influence relation for the criteria from a comparison of the *i*th dimension with the *j*th dimension. In addition, if the *i*th dimension has no influence on the *j*th dimension, then submatrix $T_{C}^{ij}=\left[0\right]$, demonstrating the independence (no influence relation) of each criterion on other criteria. Moreover, the total influence relation matrix of dimension $T_{D}$ is shown in Equation (A10).

(A10) $$T_{D}^{}={\left[\begin{array}{ccccc} t_{11}^{} & \cdots & t_{1j}^{} & \cdots & t_{1m}^{}\\ \vdots & & \vdots & & \vdots \\ t_{i1}^{} & \cdots & t_{ij}^{} & \cdots & t_{im}^{}\\ \vdots & & \vdots & & \vdots \\ t_{m1}^{} & \cdots & t_{mj}^{} & \cdots & t_{mm}^{} \end{array}\right]}_{m\times m}$$

The DANP-V model is required to identify the dimensions and criteria individually while proposing an improvement strategy; thus, drawing the INRM of dimensions and criteria should be divided into the matrix $T_{C}$ and the matrix $T_{D}$.

*Step 8: Calculate the unweighted supermatrix*$W^{\alpha }$.

Normalize the total influence relation matrix $T_{C}$ by dimensions (called “clusters”) as shown in Equation (A11).

(A11)

where $T_{C}^{\alpha }$ denotes the normalizing total influence relation matrix of criteria by dimensions, and $T_{c}^{\alpha 14}$ is derived from Equations (A12) and (A13). Similarly, $T_{c}^{\alpha mm}$ can be obtained.

(A12) $$t_{i}^{14}=\sum_{j=1}^{m_{4}}t_{ij}^{14},i=1,2,\cdots ,m_{1}$$

(A13) $$T_{c}^{\alpha 14}=\begin{array}{c} c_{11}\\ \vdots \\ c_{1i}\\ \vdots \\ c_{1m_{1}} \end{array}\begin{array}{c} \begin{array}{ccccc} c_{4}{}_{1}\; & \cdots & \;\;c_{4}{}_{j} & \;\cdots \; & \;c_{4m_{4}} \end{array}\\ \left[\begin{array}{ccccc} t_{11}^{14}/t_{1}^{14} & \cdots & t_{1j}^{14}/t_{1}^{14} & \cdots & t_{1m_{4}}^{14}/t_{1}^{14}\\ \vdots & & \vdots & & \vdots \\ t_{i1}^{14}/t_{i}^{14} & \cdots & t_{ij}^{14}/t_{i}^{14} & \cdots & t_{im_{4}}^{14}/t_{i}^{14}\\ \vdots & & \vdots & & \vdots \\ t_{m_{1}1}^{14}/t_{m_{1}}^{14} & \cdots & t_{m_{1}j}^{14}/t_{m_{1}}^{14} & \cdots & t_{m_{1}m_{4}}^{14}/t_{m_{1}}^{14} \end{array}\right] \end{array}=\begin{array}{c} \left[\begin{array}{ccccc} t_{11}^{\alpha 14} & \cdots & t_{1j}^{\alpha 14} & \cdots & t_{1m_{4}}^{\alpha 14}\\ \vdots & & \vdots & & \vdots \\ t_{i1}^{\alpha 14} & \cdots & t_{ij}^{\alpha 14} & \cdots & t_{im_{4}}^{\alpha 14}\\ \vdots & & \vdots & & \vdots \\ t_{m_{1}1}^{\alpha 14} & \cdots & t_{m_{1}j}^{\alpha 14} & \cdots & t_{m_{1}m_{4}}^{\alpha 14} \end{array}\right] \end{array}$$

According to the pairwise comparisons of the criteria and on the basis of the basic concept of ANP, the unweighted supermatrix $W^{\alpha }$ can be obtained by transposing the normalized influence relation matrix $T_{C}^{\alpha }$ by dimensions (clusters), that is, $W^{\alpha }=(T_{C}^{\alpha }{)}^{\prime }$, as shown in Equation (A14).

(A14)

*Step 9: Calculate weighted supermatrix.*

The normalized total influence-relation matrix of dimensions $T_{D}^{\alpha }$ can be obtained through the total influence-relation matrix $T^{}$ divided by $d_{i}=\sum_{j=1}^{m}t_{ij}$, i=1,2,…,m, as shown in Equation (A15).

(A15) $$T_{D}^{\alpha }={\left[\begin{array}{ccccc} t_{11}/d_{1} & \cdots & t_{1j}/d_{1} & \cdots & t_{1m}/d_{1}\\ \vdots & & \vdots & & \vdots \\ t_{i1}/d_{i} & \cdots & t_{ij}/d_{i} & \cdots & t_{im}/d_{i}\\ \vdots & & \vdots & & \vdots \\ t_{m1}/d_{m} & \cdots & t_{mj}/d_{m} & \cdots & t_{mm}/d_{m} \end{array}\right]}_{m\times m}={\left[\begin{array}{ccccc} t_{11}^{\alpha D} & \cdots & t_{1j}^{\alpha D} & \cdots & t_{1m}^{\alpha D}\\ \vdots & & \vdots & & \vdots \\ t_{i1}^{\alpha D} & \cdots & t_{ij}^{\alpha D} & \cdots & t_{im}^{\alpha D}\\ \vdots & & \vdots & & \vdots \\ t_{m1}^{\alpha D} & \cdots & t_{mj}^{\alpha D} & \cdots & t_{mm}^{\alpha D} \end{array}\right]}_{m\times m}$$

The matrix $T_{D}^{\alpha }$ and the unweighted supermatrix $W^{\alpha }$ and the weighted supermatrix ***W*** can be easily obtained by Equation (A16), where $t_{ij}^{\alpha D}$ is a scalar and $\sum_{j=1}^{m}m_{j}=n$.

(A16)

*Step 10: Limit weighted supermatrix.*

Limit the weighted supermatrix by raising it to the *z*th power until the supermatrix has converged and become a stable supermatrix. The global priority vectors, global weight $w^{g}$—which are called the IWs of DANP—are obtained, such as $\lim_{z\to \infty }{\left(W\right)}^{z}$, where z represents any number of power. By summing the IWs of each criterion in every dimension, the local weight of dimension $w_{D}^{l}$ can be obtained. Subsequently, the global weight of each criterion can be divided by the local weight of its own dimension to yield the local weight of criteria $w_{c}^{l}$.

*Step 11: Derive the positive ideal solution and the negative ideal solution replaced by the aspiration levels and the worst value to fit the current real-world situation.*

Define the best value (aspiration level) shown as $f_{j}^{aspired}$ in *j* criterion and the worst value $f_{j}^{worst}$ for all criteria. The modified approach for replacement by the aspiration level and the worst value is, as follows: The aspiration level: $f_{}^{aspired}=(f_{1}^{aspired},\ldots ,f_{j}^{aspired},\ldots ,f_{n}^{aspired})$, where $f_{j}^{aspired}$ is an aspiration level, or called the best value; The worst values: $f_{}^{worst}=(f_{1}^{worst},\ldots ,f_{j}^{worst},\ldots ,f_{n}^{worst})$, where $f_{j}^{worst}$ is a worst value. In this study, performance scores ranging from 0 to 10 (very bad ← 0, 1, 2, …, 9, 10 → very good) are used with natural language in the linguistic/semantic questionnaire; thus, the aspiration level takes the highest score of 10 and the worst value takes the value of 0. Hence, $f_{j}^{aspired}=10$ is defined as the aspiration level and $f_{j}^{worst}=0$ as the worst value to avoid choosing the best among inferior choices/options/alternatives [48].

*Step 12: Determine the mean group utility for the gap and then establish the priority improvement strategy.*

These values can be calculated using Equation (A17):

(A17) $$s_{k}=\sum_{j=1}^{n}w_{j}r_{kj}=\sum_{j=1}^{n}w_{j}(|f_{j}^{aspired}-f_{kj}|)/(|f_{j}^{aspired}-f_{j}^{worst}|)$$

where $s_{k}$ is defined as the normalized ratio (%) of distance to the aspiration level, which implies the synthesized gap of the criteria. Here, $w_{j}$ indicates the IWs for the criteria obtained from DANP.

Overall, the modified VIKOR method can also be used for the ranking and selection of alternatives and for the improvement of performance gaps of criteria [49], based on INRM produced by systematics; “stopgap piecemeal” approaches and “picking the best apple from a barrel of rotten apples” can thereby be avoided. Moreover, this method can even be used to solve any daily or single issue (i.e., a single issue, with no other alternatives) as well as for improving performance gaps when considering all perspectives (dimensions) and criteria [48,49], such as for solving the problem of this research topic.

## Appendix B. Result in Detail

As mentioned in Appendix A, we used the results of the 10 experts’ questionnaires to measure the total influence matrix ***A*** and the degrees of influence. Table A1 shows the average direct influence relation matrix $D={\left[d_{ij}\right]}_{n\times n}$ derived from the average value of the 10 experts’ questionnaires The consistency gaps of these nine questionnaires were 4.69% and were smaller than 5%, implying that the confidence level was 95.31%, which is more than 95%, as demonstrated by the following text.

**Table A1:** Average direct influence relation matrix ***D***.

*D*	*C*_11_	*C*_12_	*C*_13_	*C*_14_	*C*_21_	*C*_22_	*C*_23_	*C*_24_	*C*_25_	*C*_31_	*C*_32_	*C*_33_	*C*_41_	*C*_42_	*C*_43_	*C*_44_	*C*_51_	*C*_52_	*C*_53_	*C*_54_
*C*_11_	0.0	2.6	3	2.7	2.6	1.7	2.6	2.6	1.5	1.8	2.4	2.1	2.8	2.8	2.7	2.5	2	2.2	1.9	2.1
*C*_12_	3.1	0.0	2.7	2.6	2.7	1.6	2	1.8	1.4	1.9	2.4	2	2.6	2.7	3	2.5	2.2	2.2	1.6	1.9
*C*_13_	3.3	3.3	0.0	3.3	3.1	2	2.6	2.3	1.7	2.1	2.7	2.2	3.5	3.5	3.5	3.3	2.2	2.1	1.9	2.2
*C*_14_	3	2.7	3.3	0.0	3.2	2.2	2.7	2.6	2	2.1	2.2	1.9	3.4	3.5	3.4	3.2	2.1	2	1.5	1.8
*C*_21_	3.4	3.1	3.6	3.8	0.0	2.1	2.8	2.4	2.5	2.5	2.7	2.6	3.4	3.5	3.4	3.4	3	2.9	3.4	2.7
*C*_22_	2.3	2.2	2.5	3.1	2.1	0.0	2.7	2.5	2.1	1.3	1.5	1.5	2.7	2.6	2.7	2.5	1.4	1.4	1.3	1.4
*C*_23_	3.1	2.6	2.7	3.3	2.8	1.8	0.0	2.5	2.5	1.7	1.9	1.9	3.1	3.1	3.1	3	2.3	2.3	2.4	2.2
*C*_24_	2.8	2.3	2.4	2.7	1.7	1.8	2.3	0.0	1.3	1.9	1.9	1.8	2.7	2.4	2.8	2.1	1.6	1.5	1.7	1.8
*C*_25_	1.9	1.7	2.2	2	2.3	1.2	2.2	1.6	0.0	1.8	2	1.8	2.5	2.2	2.2	2.3	1.6	1.7	2.2	1.6
*C*_31_	1.8	2	2.1	2.1	2	1.1	1.6	1.7	1.4	0.0	2.6	2.5	2.8	2.9	3	2.2	1.6	1.4	1.3	1.5
*C*_32_	2.3	2.4	2.5	2.1	1.5	1.2	1.3	1.4	1.1	2.5	0.0	2.9	2.9	2.9	3.1	2.5	1.6	1.2	1.2	1.6
*C*_33_	1.6	1.7	1.8	1.6	1.5	1.2	1.2	1.4	1.3	2.1	2.3	0.0	2.5	2.4	2.7	2.3	1.6	1.3	1.3	1.7
*C*_41_	3.3	2.7	3	3	3.1	2.3	2.7	2.1	1.8	2.4	2.6	2.4	0.0	3.3	3.2	2.9	1.6	1.7	1.7	1.9
*C*_42_	2.6	2.6	2.8	2.8	2.4	1.8	2.1	2.1	1.5	1.8	2.1	1.9	3.1	0.0	3	2.7	1.7	1.5	1.7	1.8
*C*_43_	3	2.9	3	2.8	2.5	1.9	2.4	2.2	1.7	2.7	2.8	2.6	3.1	3.2	0.0	3.1	1.6	1.6	1.9	2.2
*C*_44_	2.4	2.4	2.4	2.1	2.8	1.4	2.3	2	1.8	1.8	1.9	2	2.4	2.4	3.1	0.0	2.1	1.8	1.8	2.2
*C*_51_	2.3	2.5	1.9	1.8	2.4	1.6	2	1.9	1.5	1.4	1.8	1.6	2.3	2.3	2.1	1.7	0.0	3.2	2.8	2.8
*C*_52_	2.6	2.1	2	2.1	2.4	1.7	2.5	2.5	2.1	1.7	2	1.8	2.2	2.5	2.7	2	2.9	0.0	3.2	3.2
*C*_53_	1.6	1.3	1.5	1.8	2.7	1.4	2.2	2	2.5	1.1	1.1	1.1	1.4	1.8	1.8	1.5	2.8	3	0.0	3
*C*_54_	2.3	2.4	2.2	1.8	2.4	1.4	1.8	2.4	1.4	2.1	2.1	2.2	2.1	2.1	2.4	2.1	2.7	2.5	2.4	0.0

Note: $\mathrm{Average}\;\mathrm{gap}-\mathrm{ratio}\;\mathrm{inconsensus}(\%)=\frac{1}{n(n-1)}\sum_{i=1}^{n}\sum_{j=1}^{n}\left(\left|d_{ij}^{H}-d_{ij}^{H-1}\right|/d_{ij}^{H}\right)\times 100\%=4.69\%<5\%$, where *n* is the number of criteria (*n* = 20), *H* is the sample of 10 experts (*H* = 10) in practical experience and significant confidence reach 95.31% (more than 95%).

Table A2, Table A3 and Table A4 show the relationship between the total influence matrix T and the degrees of influence. Table A2 illustrates the total relationships among the 20 criteria by normalization. Table A3 and A4 list the two types of information: the relationships among the five dimensions and the degree of influence of each dimension. Table A4 presents the sum of influences given or received from the degree of influence of each dimensions. Finally, Table A5 offers the sum of influences given or received from the degree of influence of each criterion within its own dimension.

**Table A2:** The normalization matrix of criteria ***N.***

*N*	*C*_11_	*C*_12_	*C*_13_	*C*_14_	*C*_21_	*C*_22_	*C*_23_	*C*_24_	*C*_25_	*C*_31_	*C*_32_	*C*_33_	*C*_41_	*C*_42_	*C*_43_	*C*_44_	*C*_51_	*C*_52_	*C*_53_	*C*_54_
*C*_11_	0.000	0.048	0.056	0.050	0.048	0.032	0.048	0.048	0.028	0.033	0.045	0.039	0.052	0.052	0.050	0.046	0.037	0.041	0.035	0.039
*C*_12_	0.058	0.000	0.050	0.048	0.050	0.030	0.037	0.033	0.026	0.035	0.045	0.037	0.048	0.050	0.056	0.046	0.041	0.041	0.030	0.035
*C*_13_	0.061	0.061	0.000	0.061	0.058	0.037	0.048	0.043	0.032	0.039	0.050	0.041	0.065	0.065	0.065	0.061	0.041	0.039	0.035	0.041
*C*_14_	0.056	0.050	0.061	0.000	0.059	0.041	0.050	0.048	0.037	0.039	0.041	0.035	0.063	0.065	0.063	0.059	0.039	0.037	0.028	0.033
*C*_21_	0.063	0.058	0.067	0.071	0.000	0.039	0.052	0.045	0.046	0.046	0.050	0.048	0.063	0.065	0.063	0.063	0.056	0.054	0.063	0.050
*C*_22_	0.043	0.041	0.046	0.058	0.039	0.000	0.050	0.046	0.039	0.024	0.028	0.028	0.050	0.048	0.050	0.046	0.026	0.026	0.024	0.026
*C*_23_	0.058	0.048	0.050	0.061	0.052	0.033	0.000	0.046	0.046	0.032	0.035	0.035	0.058	0.058	0.058	0.056	0.043	0.043	0.045	0.041
*C*_24_	0.052	0.043	0.045	0.050	0.032	0.033	0.043	0.000	0.024	0.035	0.035	0.033	0.050	0.045	0.052	0.039	0.030	0.028	0.032	0.033
*C*_25_	0.035	0.032	0.041	0.037	0.043	0.022	0.041	0.030	0.000	0.033	0.037	0.033	0.046	0.041	0.041	0.043	0.030	0.032	0.041	0.030
*C*_31_	0.033	0.037	0.039	0.039	0.037	0.020	0.030	0.032	0.026	0.000	0.048	0.046	0.052	0.054	0.056	0.041	0.030	0.026	0.024	0.028
*C*_32_	0.043	0.045	0.046	0.039	0.028	0.022	0.024	0.026	0.020	0.046	0.000	0.054	0.054	0.054	0.058	0.046	0.030	0.022	0.022	0.030
*C*_33_	0.030	0.032	0.033	0.030	0.028	0.022	0.022	0.026	0.024	0.039	0.043	0.000	0.046	0.045	0.050	0.043	0.030	0.024	0.024	0.032
*C*_41_	0.061	0.050	0.056	0.056	0.058	0.043	0.050	0.039	0.033	0.045	0.048	0.045	0.000	0.061	0.059	0.054	0.030	0.032	0.032	0.035
*C*_42_	0.048	0.048	0.052	0.052	0.045	0.033	0.039	0.039	0.028	0.033	0.039	0.035	0.058	0.000	0.056	0.050	0.032	0.028	0.032	0.033
*C*_43_	0.056	0.054	0.056	0.052	0.046	0.035	0.045	0.041	0.032	0.050	0.052	0.048	0.058	0.059	0.000	0.058	0.030	0.030	0.035	0.041
*C*_44_	0.045	0.045	0.045	0.039	0.052	0.026	0.043	0.037	0.033	0.033	0.035	0.037	0.045	0.045	0.058	0.000	0.039	0.033	0.033	0.041
*C*_51_	0.043	0.046	0.035	0.033	0.045	0.030	0.037	0.035	0.028	0.026	0.033	0.030	0.043	0.043	0.039	0.032	0.000	0.059	0.052	0.052
*C*_52_	0.048	0.039	0.037	0.039	0.045	0.032	0.046	0.046	0.039	0.032	0.037	0.033	0.041	0.046	0.050	0.037	0.054	0.000	0.059	0.059
*C*_53_	0.030	0.024	0.028	0.033	0.050	0.026	0.041	0.037	0.046	0.020	0.020	0.020	0.026	0.033	0.033	0.028	0.052	0.056	0.000	0.056
*C*_54_	0.043	0.045	0.041	0.033	0.045	0.026	0.033	0.045	0.026	0.039	0.039	0.041	0.039	0.039	0.045	0.039	0.050	0.046	0.045	0.000

**Table A3:** The total influence matrix of criteria ***T*_C._**

*T_C_*	*C*_11_	*C*_12_	*C*_13_	*C*_14_	*C*_21_	*C*_22_	*C*_23_	*C*_24_	*C*_25_	*C*_31_	*C*_32_	*C*_33_	*C*_41_	*C*_42_	*C*_43_	*C*_44_	*C*_51_	*C*_52_	*C*_53_	*C*_54_
*C*_11_	0.196	0.230	0.244	0.238	0.231	0.159	0.214	0.206	0.159	0.181	0.208	0.194	0.255	0.257	0.262	0.236	0.189	0.188	0.182	0.195
*C*_12_	0.244	0.177	0.233	0.230	0.226	0.152	0.198	0.187	0.153	0.178	0.202	0.187	0.244	0.248	0.259	0.230	0.187	0.183	0.172	0.186
*C*_13_	0.280	0.266	0.218	0.274	0.265	0.181	0.237	0.222	0.180	0.206	0.236	0.217	0.294	0.297	0.304	0.276	0.213	0.206	0.201	0.218
*C*_14_	0.268	0.249	0.268	0.209	0.259	0.180	0.232	0.221	0.180	0.200	0.221	0.205	0.285	0.289	0.294	0.267	0.205	0.199	0.189	0.205
*C*_21_	0.306	0.285	0.304	0.306	0.234	0.199	0.261	0.244	0.211	0.231	0.256	0.243	0.318	0.323	0.329	0.301	0.247	0.240	0.246	0.247
*C*_22_	0.218	0.205	0.217	0.227	0.205	0.116	0.200	0.189	0.157	0.158	0.176	0.168	0.233	0.234	0.241	0.218	0.163	0.159	0.156	0.167
*C*_23_	0.266	0.244	0.254	0.263	0.249	0.171	0.182	0.217	0.187	0.191	0.212	0.202	0.276	0.279	0.285	0.260	0.207	0.202	0.202	0.209
*C*_24_	0.224	0.204	0.213	0.217	0.195	0.146	0.190	0.142	0.141	0.166	0.181	0.171	0.230	0.227	0.240	0.208	0.165	0.159	0.161	0.172
*C*_25_	0.197	0.183	0.198	0.194	0.195	0.128	0.179	0.162	0.110	0.156	0.173	0.162	0.215	0.212	0.217	0.201	0.157	0.155	0.162	0.160
*C*_31_	0.197	0.190	0.199	0.198	0.191	0.128	0.170	0.165	0.136	0.126	0.186	0.177	0.223	0.227	0.234	0.201	0.158	0.150	0.147	0.159
*C*_32_	0.208	0.199	0.208	0.200	0.185	0.131	0.167	0.161	0.132	0.172	0.142	0.185	0.227	0.229	0.238	0.209	0.159	0.148	0.147	0.163
*C*_33_	0.176	0.168	0.176	0.171	0.166	0.118	0.148	0.145	0.122	0.149	0.165	0.118	0.198	0.199	0.208	0.185	0.143	0.135	0.134	0.148
*C*_41_	0.267	0.244	0.258	0.256	0.252	0.178	0.227	0.208	0.173	0.201	0.223	0.210	0.220	0.280	0.285	0.257	0.192	0.189	0.188	0.202
*C*_42_	0.232	0.220	0.231	0.230	0.218	0.154	0.197	0.189	0.152	0.173	0.194	0.182	0.249	0.197	0.256	0.230	0.176	0.168	0.170	0.181
*C*_43_	0.258	0.244	0.254	0.249	0.238	0.168	0.219	0.207	0.169	0.204	0.223	0.210	0.270	0.275	0.225	0.256	0.190	0.185	0.188	0.204
*C*_44_	0.224	0.212	0.220	0.213	0.220	0.144	0.196	0.184	0.154	0.170	0.187	0.180	0.232	0.235	0.252	0.178	0.179	0.170	0.169	0.185
*C*_51_	0.215	0.207	0.204	0.202	0.208	0.143	0.186	0.177	0.145	0.157	0.179	0.168	0.223	0.225	0.228	0.201	0.138	0.190	0.183	0.191
*C*_52_	0.237	0.216	0.223	0.223	0.224	0.156	0.209	0.201	0.167	0.176	0.197	0.185	0.239	0.247	0.256	0.223	0.202	0.147	0.202	0.211
*C*_53_	0.185	0.170	0.180	0.184	0.196	0.128	0.174	0.164	0.151	0.139	0.152	0.145	0.189	0.198	0.203	0.180	0.174	0.174	0.120	0.180
*C*_54_	0.218	0.209	0.212	0.204	0.210	0.141	0.185	0.188	0.145	0.172	0.187	0.181	0.223	0.226	0.236	0.211	0.188	0.180	0.177	0.144

**Table A4:** The total influence matrix of criteria ***T*_D_** and sum of influences given/received on dimensions.

*Dimensions*	*D*_1_	*D*_2_	*D*_3_	*D*_4_	*D*_5_	*o*	*r*	*o* + *r*	*o* − *r*
Workforce (*D*_1_)	0.239	0.202	0.203	0.269	0.195	1.1077	1.1098	2.2175	−0.002
Source of Funding (*D*_2_)	0.236	0.184	0.190	0.252	0.187	1.0494	0.9046	1.9539	0.1448
Application of Technology (*D*_3_)	0.191	0.151	0.158	0.215	0.149	0.8637	0.9168	1.7805	−0.053
Service Nature and Health-Care System (*D*_4_)	0.238	0.192	0.197	0.243	0.184	1.0541	1.1983	2.2524	−0.144
Norms (*D*_5_)	0.206	0.175	0.170	0.219	0.175	0.9445	0.8898	1.8343	0.0548

**Table A5:** Sum of influences given/received on criteria.

*Criteria*	*o*	*r*	*o* + *r*	*o* − *r*
Workforce matching platform (*C*_11_)	0.227	0.247	0.474	−0.020
Specialization level (*C*_12_)	0.221	0.231	0.451	−0.010
Workforce supply capacity (*C*_13_)	0.260	0.241	0.500	0.019
Labour service flexibility (*C*_14_)	0.249	0.238	0.486	0.011
Government subsidies (*C*_21_)	0.230	0.216	0.445	0.014
Stable pension (*C*_22_)	0.173	0.152	0.325	0.021
Social insurance (*C*_23_)	0.201	0.202	0.404	−0.001
Commercial insurance (*C*_24_)	0.163	0.191	0.354	−0.028
Public welfare invested donation (*C*_25_)	0.155	0.161	0.316	−0.006
Smart physiological monitoring facility (*C*_31_)	0.163	0.149	0.312	0.014
Intelligent case care system (*C*_32_)	0.166	0.164	0.331	0.002
Living-environment monitoring system (*C*_33_)	0.144	0.160	0.304	−0.016
Residential services (*C*_41_)	0.260	0.243	0.503	0.018
Case care services (*C*_42_)	0.233	0.247	0.479	−0.014
Integration of health (*C*_43_)	0.257	0.255	0.511	0.002
Health promotion (*C*_44_)	0.224	0.230	0.454	−0.006
Institutional setting standards (*C*_51_)	0.176	0.175	0.351	0.000
Operational specification (*C*_52_)	0.191	0.173	0.364	0.018
Financial specification (*C*_53_)	0.162	0.171	0.333	−0.008
Enterprise resource input specification (*C*_54_)	0.172	0.182	0.354	−0.009

Table A6 shows the supermatrix in the ANP operation, and the values of the matrix are not obtained by the questionnaire, but rather through the total influence relationship matrix. The weighted supermatrix in Table A7 is weighted by the total influence relation value of the dimensions.

**Table A6:** The un-weighted supermatrix of criteria *W*^α^.

*W* ^α^	*C*_11_	*C*_12_	*C*_13_	*C*_14_	*C*_21_	*C*_22_	*C*_23_	*C*_24_	*C*_25_	*C*_31_	*C*_32_	*C*_33_	*C*_41_	*C*_42_	*C*_43_	*C*_44_	*C*_51_	*C*_52_	*C*_53_	*C*_54_
*C*_11_	0.216	0.276	0.270	0.269	0.255	0.251	0.259	0.261	0.255	0.251	0.255	0.254	0.260	0.254	0.257	0.257	0.260	0.264	0.258	0.259
*C*_12_	0.253	0.201	0.256	0.251	0.238	0.236	0.237	0.238	0.237	0.243	0.245	0.243	0.238	0.241	0.242	0.244	0.250	0.241	0.236	0.247
*C*_13_	0.269	0.263	0.210	0.270	0.253	0.251	0.248	0.248	0.257	0.254	0.255	0.255	0.251	0.253	0.253	0.253	0.247	0.247	0.250	0.252
*C*_14_	0.262	0.260	0.264	0.210	0.255	0.262	0.256	0.253	0.251	0.252	0.245	0.248	0.250	0.252	0.248	0.246	0.243	0.248	0.256	0.242
*C*_21_	0.238	0.247	0.244	0.241	0.203	0.236	0.248	0.240	0.252	0.242	0.238	0.237	0.243	0.240	0.238	0.245	0.242	0.234	0.241	0.242
*C*_22_	0.164	0.166	0.167	0.168	0.173	0.134	0.170	0.179	0.166	0.162	0.169	0.169	0.171	0.169	0.168	0.160	0.166	0.163	0.157	0.163
*C*_23_	0.221	0.216	0.218	0.217	0.227	0.231	0.181	0.234	0.231	0.215	0.215	0.211	0.219	0.216	0.218	0.218	0.216	0.218	0.214	0.212
*C*_24_	0.213	0.204	0.205	0.206	0.213	0.218	0.216	0.175	0.209	0.209	0.208	0.208	0.201	0.208	0.207	0.204	0.206	0.210	0.202	0.216
*C*_25_	0.164	0.167	0.166	0.168	0.184	0.181	0.186	0.173	0.142	0.172	0.170	0.175	0.167	0.167	0.169	0.172	0.169	0.175	0.186	0.167
*C*_31_	0.311	0.313	0.313	0.320	0.317	0.314	0.315	0.320	0.317	0.258	0.344	0.345	0.318	0.315	0.320	0.316	0.312	0.315	0.318	0.319
*C*_32_	0.357	0.357	0.358	0.352	0.351	0.351	0.351	0.349	0.352	0.381	0.284	0.382	0.351	0.353	0.350	0.348	0.355	0.353	0.349	0.347
*C*_33_	0.332	0.330	0.329	0.328	0.332	0.334	0.334	0.330	0.330	0.362	0.371	0.272	0.331	0.331	0.330	0.336	0.333	0.332	0.332	0.335
*C*_41_	0.252	0.249	0.251	0.251	0.250	0.252	0.251	0.254	0.254	0.252	0.251	0.251	0.211	0.267	0.263	0.259	0.254	0.248	0.246	0.249
*C*_42_	0.255	0.253	0.254	0.255	0.254	0.252	0.253	0.251	0.251	0.256	0.254	0.251	0.269	0.212	0.268	0.262	0.257	0.256	0.257	0.252
*C*_43_	0.259	0.264	0.260	0.259	0.259	0.260	0.259	0.265	0.257	0.264	0.264	0.264	0.274	0.274	0.219	0.281	0.260	0.266	0.263	0.264
*C*_44_	0.234	0.234	0.236	0.235	0.237	0.235	0.236	0.230	0.238	0.228	0.231	0.234	0.246	0.247	0.250	0.198	0.229	0.231	0.234	0.235
*C*_51_	0.251	0.257	0.254	0.257	0.252	0.253	0.252	0.251	0.247	0.256	0.258	0.256	0.249	0.253	0.247	0.255	0.197	0.265	0.268	0.272
*C*_52_	0.250	0.251	0.246	0.249	0.245	0.247	0.246	0.242	0.244	0.244	0.240	0.241	0.245	0.242	0.241	0.242	0.271	0.193	0.268	0.262
*C*_53_	0.241	0.236	0.240	0.237	0.251	0.242	0.247	0.245	0.256	0.240	0.238	0.239	0.244	0.245	0.246	0.240	0.260	0.265	0.186	0.257
*C*_54_	0.259	0.256	0.260	0.257	0.252	0.258	0.255	0.262	0.253	0.259	0.264	0.265	0.262	0.261	0.266	0.263	0.272	0.277	0.278	0.209

**Table A7:** The weighted supermatrix of criteria *W.*

*W*	*C*_11_	*C*_12_	*C*_13_	*C*_14_	*C*_21_	*C*_22_	*C*_23_	*C*_24_	*C*_25_	*C*_31_	*C*_32_	*C*_33_	*C*_41_	*C*_42_	*C*_43_	*C*_44_	*C*_51_	*C*_52_	*C*_53_	*C*_54_
*C*_11_	0.047	0.060	0.058	0.058	0.057	0.057	0.058	0.059	0.057	0.056	0.056	0.056	0.059	0.057	0.058	0.058	0.057	0.057	0.056	0.056
*C*_12_	0.055	0.043	0.055	0.054	0.053	0.053	0.053	0.054	0.053	0.054	0.054	0.054	0.054	0.054	0.055	0.055	0.054	0.052	0.051	0.054
*C*_13_	0.058	0.057	0.045	0.058	0.057	0.056	0.056	0.056	0.058	0.056	0.056	0.056	0.057	0.057	0.057	0.057	0.054	0.054	0.054	0.055
*C*_14_	0.057	0.056	0.057	0.045	0.057	0.059	0.058	0.057	0.057	0.056	0.054	0.055	0.057	0.057	0.056	0.056	0.053	0.054	0.056	0.053
*C*_21_	0.044	0.045	0.044	0.044	0.036	0.042	0.044	0.042	0.044	0.042	0.042	0.041	0.044	0.044	0.043	0.045	0.045	0.043	0.045	0.045
*C*_22_	0.030	0.030	0.030	0.031	0.030	0.023	0.030	0.032	0.029	0.028	0.030	0.029	0.031	0.031	0.031	0.029	0.031	0.030	0.029	0.030
*C*_23_	0.040	0.039	0.040	0.040	0.040	0.041	0.032	0.041	0.041	0.038	0.038	0.037	0.040	0.039	0.040	0.040	0.040	0.040	0.040	0.039
*C*_24_	0.039	0.037	0.037	0.038	0.037	0.038	0.038	0.031	0.037	0.036	0.036	0.036	0.037	0.038	0.038	0.037	0.038	0.039	0.037	0.040
*C*_25_	0.030	0.030	0.030	0.031	0.032	0.032	0.033	0.030	0.025	0.030	0.030	0.031	0.030	0.030	0.031	0.031	0.031	0.032	0.034	0.031
*C*_31_	0.057	0.057	0.057	0.059	0.057	0.057	0.057	0.058	0.057	0.047	0.063	0.063	0.059	0.059	0.060	0.059	0.056	0.057	0.057	0.057
*C*_32_	0.065	0.065	0.066	0.065	0.063	0.064	0.063	0.063	0.064	0.070	0.052	0.070	0.066	0.066	0.065	0.065	0.064	0.064	0.063	0.062
*C*_33_	0.061	0.060	0.060	0.060	0.060	0.060	0.060	0.060	0.060	0.066	0.068	0.050	0.062	0.062	0.062	0.063	0.060	0.060	0.060	0.060
*C*_41_	0.061	0.060	0.061	0.061	0.060	0.061	0.060	0.061	0.061	0.063	0.062	0.062	0.049	0.062	0.061	0.060	0.059	0.058	0.057	0.058
*C*_42_	0.062	0.061	0.062	0.062	0.061	0.061	0.061	0.060	0.060	0.064	0.063	0.062	0.062	0.049	0.062	0.060	0.060	0.059	0.060	0.058
*C*_43_	0.063	0.064	0.063	0.063	0.062	0.063	0.062	0.064	0.062	0.066	0.066	0.066	0.063	0.063	0.051	0.065	0.060	0.062	0.061	0.061
*C*_44_	0.057	0.057	0.057	0.057	0.057	0.057	0.057	0.055	0.057	0.057	0.057	0.058	0.057	0.057	0.058	0.046	0.053	0.054	0.054	0.055
*C*_51_	0.044	0.045	0.045	0.045	0.045	0.045	0.045	0.045	0.044	0.044	0.045	0.044	0.043	0.044	0.043	0.044	0.036	0.049	0.050	0.050
*C*_52_	0.044	0.044	0.043	0.044	0.044	0.044	0.044	0.043	0.043	0.042	0.042	0.042	0.043	0.042	0.042	0.042	0.050	0.036	0.050	0.049
*C*_53_	0.042	0.041	0.042	0.042	0.045	0.043	0.044	0.044	0.046	0.041	0.041	0.041	0.042	0.043	0.043	0.042	0.048	0.049	0.034	0.048
*C*_54_	0.045	0.045	0.046	0.045	0.045	0.046	0.045	0.047	0.045	0.045	0.046	0.046	0.046	0.045	0.046	0.046	0.050	0.051	0.052	0.039
